# Underage Binge Drinking Adolescents: Sociodemographic Profile and Utilization of Family Doctors

**DOI:** 10.5402/2013/728730

**Published:** 2013-02-07

**Authors:** Esme Fuller-Thomson, Matthew P. Sheridan, Cathy Sorichetti, Rukshan Mehta

**Affiliations:** ^1^Faculty of Social Work, University of Toronto, 246 Bloor Street Westt, Toronto, ON, Canada M5S 1A1; ^2^Peterborough Area Program, Kinark Child and Family Services, Suite 200, 500 Hood Road, Markham, ON, Canada L3R 9Z3

## Abstract

*Context*. Binge drinking (more than five drinks on one occasion) is a major public health problem among teenagers in the US, Canada, and Europe. Negative outcomes to binge drinking include alcohol related injuries and accidental death. Family physicians are the main point of contact between binging adolescents and the health care system. *Design and Setting*. This study was based on a secondary analysis of 6,607 respondents aged 15–17 from the regionally representative data acquired through the Canadian Community Health Survey 1.1. *Results*. According to our findings, one in every eight teens aged 15–17 binge drank monthly. The odds of binge drinking were higher among males, Whites, those living away from parents, teens who reported a decline in health status, and those experiencing back problems and depression. Smoking status was strongly associated with the binge drinking behavior. Three-quarters of binge drinking adolescents had seen their family doctor in the past year but only one in ten had spoken with any health professional about a mental health issue. *Conclusions*. Family physicians need to screen their adolescent patients for binge drinking in order to provide timely and effective interventions. Awareness of the profile of binge drinkers could improve the accuracy of targeting and outreaching strategies.

## 1. Introduction

 Binge drinking, defined as consuming five or more drinks on one occasion, is a common occurrence among many teen patients of family practitioners. Despite the fact that these teens are under the legal age for purchasing alcohol, many adolescents engage in underage drinking, in general, and binge drinking, in particular. An estimated 19% of underage American adolescents 12–20 years of age were involved in binge drinking behavior at least once per month [1, 2].

There are many negative short-term and long-term outcomes of binge drinking. Driving while intoxicated and subsequent alcohol related motor vehicle accidents are higher among adolescent binge drinkers [3]. Other alcohol related injuries and deaths such as choking on one's vomit and alcohol poisoning are also consequences of binge drinking [2]. Adolescent binge drinkers are also more likely to be involved in physical fighting and to be injured and/or injure others when compared to nonbingers [4].

Binge drinking among adolescents is also associated with earlier initiation of sex, higher rates of sexual activity, more sexual partners, and increased prevalence of unprotected sex [5]. Binge drinking adolescent women also experience higher rates of sexual victimization, teen pregnancy, and parenting [6–8]. Even adolescent girls who consume moderate amounts of alcohol may experience disrupted growth and puberty [8].

By their early 20s, individuals who had been underage binge drinkers have higher rates of health problems and unsafe health behaviors such as smoking, unprotected sex, and drunk driving [6, 7, 9]. Furthermore, they are much more likely to have not completed high school [10] or college [11], and to be selling drugs [11]. Binge drinking is associated with subsequent alcoholism, adult binge drinking, and substance abuse problems [12–14]. 

The monetary and economic cost of underage drinking is estimated to be 62 billion dollars annually [15]. A constellation of risk factors are predictors of adolescents becoming binge drinkers: parental alcoholism, alienation, greater susceptibility to peer pressure, peers who are drinkers and/or taking illegal drugs, less parental monitoring, and lower academic functioning [16–18]. Males and White adolescents are more likely to be binge drinkers than are females and visible minority members. 

 Adolescents are more likely to see family doctors than any other professional health care provider. Little information is known about how many doctors discuss binge drinking with their adolescent patients. Brief interventions by family physicians to reduce high risk drinking have been shown to result in significant long-term reductions in binge drinking behavior [19]. 

There are gaps in the extant literature on binge drinking among underage adolescents. In particular, there is the need for gender-specific analyses using representative community samples from countries other than the USA or UK. In order to address these gaps, this study's objective was to determine the gender-specific demographic, health and health care utilization profile of binge drinking Canadian youth aged 15 through 17. 

## 2. Methods

### 2.1. Sample

This study uses data from the 2000-2001 Canadian Community Health Survey 1.1 [20]. The CCHS 1.1 is a national cross-sectional survey of a representative sample of individuals aged 12 years and older [20]. The data were gathered by Statistics Canada, the Canadian equivalent of the U.S. Census Bureau, using a multistage stratified cluster-sampling design. Face-to-face interviews were conducted with 88% of the sample and telephone interviews were conducted with the remaining 12% [20]. The overall response rate was 84.7%. Extensive details on the surveys' methodologies and sampling strategies are provided elsewhere (see [20]).

In Canada, the legal drinking age is either 18 or 19, dependent on the province. We chose to focus on 15 to 17 year olds because these individuals were below the legal drinking age in every province. There were 6685 respondents aged 15 to 17. Of these 78 had missing data on the binge drinking variable; therefore, the final sample size for our study was 6607. The logistic regression analyses included only the 6,198 respondents who had complete data on all the variables included in the model. Because of the CCHS's stratified, multistage sampling design, all data are weighted using a weighting variable Statistics Canada designed to address household nonresponse and oversampling [20].

### 2.2. Measures

#### 2.2.1. High-Risk Drinking Behaviors

Respondents were identified as *binge drinkers* if they reported that, at least one time per month during the past 12 months, they had 5 or more drinks on one occasion. Individuals who reported having 5 or more drinks at least once a month during the last 12 months were asked additional questions to determine alcohol dependence. *Alcohol dependence* was assessed through the Composite International Diagnostic Interview-Short Form (CIDI-SF) developed by Kessler et al. to operationalize both Criterion A and Criterion B of the DSM-III-R diagnoses for psychoactive substance use disorder [21]. Sensitivity of the CIDI-SF was 93.6% and specificity was 96.2% with a total classification accuracy of 95.8% for alcohol dependence in comparison to CIDI [21]. Respondents were classified as probably alcohol dependent if they had a probability of 85% or higher on the scale, which means that the respondent reported at least three of the following symptoms of alcohol dependence in the past month: being drunk or hungover while at work or school or while taking care of children, engaging in a risk-taking behavior while drunk or hungover, having psychological problems related to alcohol use, experiencing a persistent desire for alcohol, drinking too much or too long, or experiencing increased tolerance [21].

#### 2.2.2. Demographic Characteristics

The following demographic variables were investigated: (1) *currently attending school *or college (yes/no); (2) *race *(self-report: White versus non-White); (3) *living arrangement* (living with 2 parents, living with single parent, unattached and not living with a parent, other); (4) *income adequacy *(low/middle/high) was based on total household income adjusted for the number of people living in the household; (5) *food insecurity *was defined as sometimes or often not having enough food to eat because of a lack of money.

#### 2.2.3. Health Indicators

*Self-reported health status* was based on individual's response when asked, “In general, would you say your health is excellent, very good, good, fair, poor.” Response categories were dichotomized into excellent, very good, or good, versus fair or poor. *Self-reported health decline* was assessed through respondents' answers to the question “Compared to one year ago, how would you say your health is now?” The responses were dichotomized into much better/somewhat better now than a year ago or the same versus somewhat worse or much worse. Participants were asked two questions on limitations due to pain. If individuals answered no to the first question asking if they were usually free of pain and discomfort, they were asked a subsequent question on how many *activities were limited by pain* or discomfort prevented (none, a few, some, most). From these two questions, dichotomous categories were created (usually no pain or discomfort or pain prevents no activities versus pain prevents few activities, some activities, or most activities). The respondents were asked if a health professional had diagnosed them with any of the following 17 *chronic conditions* which had to be of six months or longer duration: fibromyalgia, arthritis or rheumatism, cataracts, chronic bronchitis, emphysema or chronic obstructive pulmonary disease, diabetes, epilepsy, heart disease, inflammatory bowel disease, cancer, stomach or intestinal ulcers, effects of a stroke, glaucoma, and chronic fatigue syndrome, asthma, back problems (excluding fibromyalgia and arthritis), and migraine headaches. These conditions were subsequently summed and categorized as no chronic conditions, 1 chronic condition, and 2 or more chronic conditions. Only the latter three conditions were present in at least 5% of the 15–17-year-old sample. These three conditions were therefore examined separately.

Depression was assessed using the Composite International Diagnostic Interview short form [21], which was designed to operationalize Criteria A through C of the DSM-III-R diagnosis of major depressive episode. The CIDI-SF has been shown to have very high sensitivity, specificity, and classification accuracy for a major depressive episode in comparison to CIDI [21]. The respondents were considered to experience depression if their depression score was 5 or more (90% likelihood of a positive diagnosis of major depressive episode). Certain health regions opted to include the following question on *suicidal ideation, *“Have you ever seriously considered committing suicide or taking your own life.” The sample size for this question is considerably smaller than the full data.

Four Medical Outcomes Survey (MOS) subscales on social support were used. These scales have been shown to be both reliable and valid [22]. The first self-report subscale, *affection,* assesses the amount of affection received by the respondent (e.g., whether there is someone who loves them, hugs them, and makes them feel wanted). Another MOS subscale assesses the availability of *tangible social support* (e.g., whether there is someone who, if asked, could help them if they were confined to bed or needed to be taken to the doctor). The third MOS subscale evaluated *emotional and informational support* (e.g., whether there was someone to listen and advise them in a crisis or to understand their problems) [22]. The self-esteem scale measures positive self-regard and is a subset of the Rosenberg self-esteem scale. 

#### 2.2.4. Health Care Utilization

The extent to which respondents used family doctors was determined by the question: “Not counting when you were an overnight patient (in the hospital), in the past 12 months, how many times have you seen or talked to on the telephone to a *family doctor *about your physical, emotional or mental health.” Response categories were never versus 1 or more times. *Number of mental health visits *to any professional was determined through the question: “How many times in the past 12 months… have you seen, or talked on the telephone, to a health professional about your emotional or mental health?” Responses were categorized as no visits, 1–3 visits, 4 or more visits. Respondents were then asked to identify all the types of health professionals, including family doctors, whom they had seen or spoken to about their emotional or mental health. 

*Unmet mental health care need *(yes/no) was assessed through two questions: (1) during the past 12 months, was there ever a time when you felt that you needed health care but you did not receive it? (2) thinking of the most recent time, what was the type of care that was needed: Treatment of an emotional or mental health problem? *Use of self-help groups* was determined through the question asking if they had “attended a self-help group such as Alcoholics Anonymous or a cancer support group in the past 12 months”.

#### 2.2.5. Statistical Analysis Strategy

The analysis of the data essentially was to compare the demographic, health and health care utilization characteristics associated with binge drinking monthly among male adolescents (*n* = 528 binge drinkers, 2,783 nonbinge drinkers) and female adolescent aged 15–17 (*n* = 361 binge drinkers, 2935 non-binge drinkers). Using SPSS version 20, the authors conducted frequencies, chi-square tests for categorical data, and two-way ANOVAs for continuous variables (e.g., 4 types of social support and self-esteem) and logistic regression analysis. 

## 3. Results

An estimated 13.5% of 15–17-year-old teenagers were binge drinking at least one time per month. This number represents one in every 8 adolescents in this age cohort. Males were more likely to binge drink at least once monthly as compared to females (16.0% versus 10.9%). Although binge drinking was rarer among females than males, binging females were more likely to be classified as alcohol dependent than binging males. As a result, a comparable percentage of Canadian 15–17-year-old males and females were found to be alcohol dependent (2.3% of men, 2.2% of women). 

Despite these high rates of heavy drinking, only one in ten (9%) binge drinking adolescents had spoken with any health professional about a mental health or emotional issue in the previous year. Fewer than 5% of binge drinking teens had attended a self-help group. 

Three out of every four binge drinking adolescents (74%) had seen or talked on the telephone with their family physician in the past year, suggesting that family doctors are a major source of health care for this population. Unfortunately, only 1% of these underage binge drinkers had spoken with their family doctor about a mental health or emotional issue in the preceding year. 

Many demographic factors were significantly associated with binge drinking. Both female and male binge drinkers were more likely to be White (please see Table 1). Both genders were less likely to be enrolled in school or to be living with parents. Females who lived in single parent families were more likely to binge drink than those in two parent families. Males and females who lived on their own had particularly high binge drinking rates. Males from richer households binged more than those from poorer households. However, among females the opposite relationship existed: poorer females binge drank more than their richer peers. Women who were sometimes without enough food to eat because of a lack of money were much more likely to binge than those with adequate food. This factor was not associated with binge drinking among males.

For both genders, factors related to physical health characteristics were related to binge drinking rates. Males and females who reported poor or fair health had much higher rates of binge drinking than did those in good or excellent health. Those who had experienced a health decline in the previous year were likely more to be binge drinkers than those who had not. Male and female adolescents who had activities prevented by pain also had much higher rates of binge drinking than did individuals who were not constrained by pain. Among women, but not men, those with more chronic conditions were more likely to binge drink, and back problems and migraines were both associated with a higher prevalence of binging. Although having more chronic conditions, in general, was not associated with binge drinking among males, those with back problems were more likely to binge drink and those with asthma were less likely to binge. 

Health behaviors were associated with binge drinking behaviors. For both genders, smokers and those who were sexually active had a much higher prevalence of binge drinking. Men who were binge drinkers were more physically active than non-bingers. There was no relationship between physical activity and binge drinking for women.

The two-way ANOVA analyses revealed that binge drinkers had significantly lower levels than non-binge drinking adolescents of three types of social support (emotional/information support, affection and tangible social support; *P* < .001) and self-esteem (*P* = .01). In addition, there were two interaction effects between binge drinking status and gender. There was a much wider difference in levels of emotional/information support between binge drinking and non-binge drinking males than between their female counterparts (*P* < .001). There was a statistical trend (*P* = .07) indicating that opposite was true for self-esteem: female binge drinkers had very low self-esteem compared to non-binging women, while the gap in self-esteem levels between binging and non-binging men was quite small. Binge drinking status was not associated with the level of positive social interactions.

There were several factors which were much more prevalent among binge drinkers than non-bingers. Of particular relevance to family physicians, binge drinking adolescents had much higher rates of suicidal ideation than non-bingers (10.2% versus 3.0%, *P* < .001). Teens who were binge drinkers also had a higher prevalence of depression than non-binging adolescents (13.1% versus 6.1%; *P* < .001). 

The logistic regression analysis (please see Table 2) indicated that males had twice the odds of binge drinking and teens not attending school or university had almost 50% higher odds of binge drinking compared to their school-attending peers. Whites had four times the odds of binge drinking in comparison to non-Whites. In comparison to those living with two parents, those living on their own had more than two times the odds of binge drinking at least monthly. Teens who reported a decline in health status in the preceding year had 85% higher odds of binge drinking in comparison to those whose health had remained the same or improved. Adolescents with back problems had 87% higher odds of binge drinking than their peers without back problems. Depressed teens had 62% higher odds of binge drinking than those who were not depressed. Adolescents who smoked had more than five times the odds of binge drinking in comparison to nonsmokers.

The Nagelkerke R-square statistic (see Table 3) indicates that demographic characteristics explained 7.5% of the variability in binge drinking. Physical health characteristics contributed an additional 2.4% and depression explained an additional 0.8% of the variability in binge drinking. When smoking status was added to the equation, it explained an additional 11.3% of the variability in binge drinking, doubling the total amount explained to 22.2%.

## 4. Discussion

Approximately one of every eight teens aged 15–17 was binge drinking at least monthly. The finding that one in fifty underage adolescents was classified as “alcohol dependent” is very worrisome. The odds of binge drinking were higher among males, Whites, those living away from parents, teens who reported a decline in health status, and those experiencing back problems and depression. Smoking status was strongly associated with binge drinking behavior. Three-quarters of all binge drinking teens had seen or spoken with a family physician at least once in the past year although fewer than 10% have consulted with any health professional about mental health or emotional issues during that time period, suggesting the need for improved outreach and screening. Knowledge of those factors associated with binge drinking in underaged adolescents could help family doctors and other health professionals target those most at risk for binge drinking.

 Family doctors need to be aware of the health problems associated with an elevated prevalence of binge drinking. In the bivariate analyses, we found that back problems and pain were correlated with alcohol abuse as has been previously documented [23, 24]. Research proposes that alcohol may in some cases be used to self-medicate when feeling physical pain [23, 24], which may also support our finding that those in declining health had almost twice the odds of binge drinking. Similar to the findings of Jukkala and colleagues, we observed that a lack of social support was associated with binge drinking [25]. Many of our other findings were also in keeping with previous studies, such as our data suggesting non-Whites binge drink less than White adolescents (e.g., [26]). In contrast to previous findings, males with lower socioeconomic status were less likely to engage in binge drinking, possibly due to prohibitive costs in Canada, where alcohol is heavily taxed [27, 28].

Family doctors should be particularly cognizant that binge drinking may be a marker for other negative outcomes including suicidal ideation and depression, both of which were found at a much higher prevalence among binge drinking teens than nonbinging adolescents. According to Miller and colleagues, young girls who engage in binge drinking and heavy alcohol use are twice as likely to attempt suicide compared to girls who do not drink [29]. Alcoholism is also associated with risky sexual behaviour, and people who exchange sex for food due to extreme poverty are also more likely to use alcohol [30]. Lower social support and self-esteem play a major role in alcohol abuse and binge drinking among women [31].

 Bonomo and her colleagues conclude that prevention and early intervention strategies have a significant impact on the trajectories of early adulthood alcohol use [12]. Grossberg et al. used a brief intervention in a health care setting in which physicians delivered 2 short counseling sessions over a 4-week period to binge drinking young adults [19]. During the 4-year follow-up period it was found that the young adults who received the intervention experienced a long-term reduction in alcohol use, including binge drinking. Such an intervention could be done in conjunction with other school and community based programs to decrease binge drinking in underaged individuals. Other promising programs include educating parents about the risks of supplying alcohol to teenagers [32], enhancing penalty enforcement for licensed liquor sellers who sell to minors [33], implementing life skills training with inner city youth while educating them on the risk of binge drinking [34], and providing multi-year curricula starting in middle school to address binging behavior, including peer participation and task force activities involving the community [35].

Unfortunately, our analyses did not allow us to evaluate many aspects of the adolescent's environment. Other researchers have suggested that early onset of drinking is strongly related to social factors such as availability of alcohol and peer influence [14].

The high prevalence of binge drinking found in this research underlines the importance of family physicians actively screening their adolescent patients for binge drinking as is routinely done with smoking and other risky behaviors. Our findings indicate the vast majority of binge drinking adolescents are not proactively turning to their family doctors with this issue. The profile of those at risk will help physicians assess need and create a course of action to help their patients recover from such hazardous behavior. Promising interventions include short counseling sessions [19].

## Figures and Tables

**Table 1:** Gender specific demographic, health and health care utilization characteristics by binge drinking status. Source: 15–17 years old subsample of the Canadian Community Health Survey 1.1 [20].

Variable	Male total *n*	Male % Binge drinker	Male *P* value	Female total *n*	Female % Binge drinker	Female *P* value
Gender^1^	3311	16.0%		3296	10.9%	

*Demographic characteristics *						
Currently attending						
School/college						
Yes	2960	15.1%		3021	10.2%	
No	343	23.6%	<.001	256	20.3%	<.001
Race						
White	2681	18.7%		2719	12.4%	
Visible minority	586	4.3%	<.001	524	3.4%	<.001
Living arrangement						
Child with 2 parents	2224	15.3%		2267	8.5%	
Child with single parent	577	15.9%		532	16.4%	
Unattached	84	31.0%	.001	115	20.9%	<.001
Other	367	18.0%		346	15.3%	

*Socioeconomic characteristics *						
Income adequacy						
Low	294	12.2%		346	14.5%	
Middle	676	13.8%		671	12.2%	
High	1789	17.0%	.05	1700	10.5%	.04
Missing data	553	17.2%		580	8.8%	
Not enough to eat due to lack of money						
Always enough food	3020	16.0%		2971	10.4%	
Sometimes/often not enough	254	15.4%	.78	277	18.1%	<.001

*Physical health characteristics *						
Self-reported health status						
Fair/poor	152	23.7%		201	19.9%	
Excellent/good	3160	15.6%	.008	3093	10.4%	<.001
Self-reported health decline (1 yr)						
Much better/same	3125	15.2%		3015	9.6%	
Somewhat/much worse	187	28.9%	<.001	281	26.0%	<.001
Activities prevented by pain						
No activity prevented	3251	15.6%		3122	10.6%	
Some activity prevented	58	34.5%	<.001	172	17.4%	.005
Chronic conditions						
No chronic conditions	2558	15.9%		2279	9.4%	
One chronic condition	631	15.8%	0.79	784	13.8%	<.001
Two+ chronic condition	121	18.2%		227	17.2%	
Asthma						
No	2873	16.6%		2828	10.6%	
Yes	438	11.6%	.008	467	13.3%	.08
Back problems (excluding arthritis)						
No	3139	15.6%		2999	10.0%	
Yes	173	22.0%	.026	297	20.2%	<.001
Migraines						
No	3148	15.7%		2971	10.5%	
Yes	163	20.9%	.08	325	15.1%	.01

*Health behaviors *						
Sexual activity^2^						
Has had sexual intercourse	326	32.5%		338	23.7%	
Never had sexual intercourse	1273	8.6%	<.001	1226	5.0%	<.001
Physical activity level^2^						
Active	1537	20.3%		1051	12.9%	
Moderate	641	15.3%	<.001	754	10.7%	.37
Inactive	705	14.0%		1179	12.0%	
Smoking status						
Never smoked	2054	7.5%		1993	3.0%	
Former or current smoker	1243	30.0%	<.001	1296	23.2%	<.001

^1^ *P* < .001 for gender by binge drinking chi-square.

^2^Not all health districts included these questions in their survey; therefore, the sample size is lower.

**Table 2:** Logistic regression analysis of binge drinking status by demographic, health characteristics (*N* = 6,198). Source: 15–17 years old subsample of the Canadian Community Health Survey 1.1 [20].

	Odds ratios	95% confidence interval
*Demographic characteristics *		
Gender^3^		
Female	1.00	Reference
Male	**1.98**	**(1.68, 2.34)**
Currently attending school/college		
Yes	1.00	Reference
No	**1.47**	**(1.17, 1.86)**
Race (self-report)		
Non-White	1.00	Reference
White	**4.33**	**(3.06, 6.13)**
Living Arrangement		
Child with 2 parents	1.00	Reference
Child with single parent	1.15	(0.93, 1.42)
Unattached	**2.28**	**(1.53, 3.39)**
Other	**1.33**	**(1.04, 1.70)**

*Socioeconomic characteristics *		
Income adequacy		
Low	1.00	Reference
Middle	1.16	(0.84, 1.60)
High	1.35	(1.00, 1.82)
Missing data	1.26	(0.90, 1.77)
Not Enough to eat due to lack of money		
Always enough food	1.00	Reference
Sometimes/often not enough	1.05	(0.79, 1.41)

*Physical health characteristics *		
Activities prevented by pain		
No activity prevented	1.00	Reference
Some activity prevented	1.35	(0.92, 1.98)
Chronic conditions		
No chronic conditions	1.00	Reference
One chronic condition	0.79	(0.54, 1.16)
Two+ chronic condition	0.53	(0.25, 1.11)
Self-reported health decline (1 yr)		
Much better/same	1.00	Reference
Somewhat/much worse	**1.85**	**(1.44, 2.38)**
Asthma		
No	1.00	Reference
Yes	1.08	(0.71, 1.64)
Back problems (excluding arthritis)		
No	1.00	Reference
Yes	**1.87**	**(1.20, 2.92)**
Migraines		
No	1.00	Reference
Yes	1.40	(0.90, 2.18)

*Mental health characteristics *		
Depression		
Not depressed	1.00	Reference
Depressed	**1.62**	**(1.25, 2.10)**

*Health behaviors *		
Smoking status		
Never smoked	1.00	Reference
Former or current smoker	**5.58**	**(4.70, 6.65)**

^3^ *P* < .001 for gender by binge drinking chi-square.

**Table 3:** Model Characteristics of the Logistic Regression Analyses.

Model summary	Change in chi-square due to these variables	*P* value of chi-square	Change in Nagelkerke *R*-square due to these variables
Demographic characteristics	261.6	*P* < .001	7.5%
Socioeconomic characteristics	6.2	*P* = .19	0.2%
Physical Health characteristics	86.0	*P* < .001	2.4%
Depression	30.7	*P* < .001	0.8%
Smoking status	422.6	*P* < .001	11.3%
Full model	807.1	*P* < .001	22.2%
